# Activation of phagocytosis by immune checkpoint blockade

**DOI:** 10.1007/s11684-018-0657-5

**Published:** 2018-07-30

**Authors:** Chia-Wei Li, Yun-Ju Lai, Jennifer L. Hsu, Mien-Chie Hung

**Affiliations:** 1 Department of Molecular and Cellular Oncology, The University of Texas MD Anderson Cancer Center, Houston, TX 77030, USA; 2 Department of Neurology, McGovern Medical School, The University of Texas Health Science Center at Houston, Houston, TX 77030, USA

**Keywords:** CD47, PD-1, PD-L1, immunotherapy, TAM, phagocytosis, macrophage

## Abstract

Inhibition of macrophage-mediated phagocytosis has emerged as an essential mechanism for tumor immune evasion. One mechanism inhibiting the innate response is the presence of the macrophage inhibitory molecule, signal regulatory protein-α (SIRPα), on tumor-associated macrophages (TAMs) and its cognate ligand cluster of differentiation 47 (CD47) on tumor cells in the tumor microenvironment. On the basis of a recently discovered programmed death protein 1 (PD-1) in TAMs, we discuss the potential inhibitory receptors that possess new functions beyond T cell exhaustion in this review. As more and more immune receptors are found to be expressed on TAMs, the corresponding therapies may also stimulate macrophages for phagocytosis and thereby provide extra anti-tumor benefits in cancer therapy. Therefore, identification of biomarkers and combinatorial therapeutic strategies, have the potential to improve the efficacy and safety profiles of current immunotherapies.

## Introduction

The field of immuno-oncology has dramatically reshaped the landscape of cancer therapy since the development of antibodies that block immune checkpoint proteins, which activate anti-tumor immunity by enhancing T cell cytolytic activity [1]. To date, dozens of immune checkpoint blockades have been developed for clinical trials in treating leukemia, lymphoma, and solid tumors [2,3]. In particular, antibodies targeting immune checkpoint protein programmed death protein 1 (PD-1) and programmed death ligand 1 (PD-L1) have been approved by the Food and Drug Administration (FDA) to treat 25 types of cancers in over 120 000 patients [4]. Despite their clinical success in producing durable responses, the overall response rate of immunotherapy is 15%—20% [5]. Many combinatorial strategies were thus rationally designed and tested in the clinic in attempt to enhance the therapeutic outcome of cancers [3]. Moreover, issues such as missing targets, intrinsic/acquired resistance, hyper-progressive diseases, the lack of reliable biomarkers, autoimmune diseases, and neurotoxicity, have now become the new challenges awaiting further investigation [6–8]. Therefore, furthering our understanding of the mechanisms underlying cell-cell interaction may help identify responsive cohorts of patients and enhance the response rate of immunotherapy. Immune defense is typically divided into two categories: innate and adaptive response. Innate immunity refers to the nonspecific recognition that reacts immediately after antigen exposure whereas adaptive immunity involves more complex processes, including antigen recognition and T cell activation in order to eliminate the specific antigen. The field of cancer immunotherapy has primarily focused on the molecular interaction between cancer and the effector T cells in the tumor microenvironment; however, targeting the signaling that allow cluster of differentiation 47 (CD47)-mediated inhibition of macrophage engulfment has emerged as a new type of immunotherapy strategy [9]. Virtually expressed in all types of cancers, CD47 is a transmembrane molecule that engages with signal regulatory protein-α (SIRPα) on the dendritic cells and macrophages [10]. Through overexpression of CD47 on their surface, cancer cells defend themselves against phagocytosis by macrophages. High expression of CD47 has been shown to correlate with poorer disease survival in many cancer types, including acute myeloid leukemia [11], breast carcinoma [12,13], esophageal carcinoma [14], and gastric cancer [15]. Low expression of CD47 is correlated with positive disease outcome of ovarian carcinoma [16]. Indeed, pathological evidence has consistently shown that expression of CD47 is a pro-tumorigenic factor. In a therapeutic setting, monoclonal antibodies that block the interaction between CD47 and SIRPα robustly reawakens the innate immunity in mice [11]. Furthermore, combining anti-CD47 with anti-PD-1 induced stronger antitumor immunity than anti-CD47 alone [17]. Currently, ten clinical trials are underway to test the efficacy of anti-CD47 agents (Hu5F9-G4, TTI-621, or CC-90002) as monotherapy or in combination with chemotherapy or target therapy to treat acute myeloid leukemia, colorectal cancer, solid tumor, and non-Hodgkin’s lymphoma [18]. Because CD47 is also expressed in normal tissues, toxicity has been observed in the pre-clinical study, but the adverse events are manageable [11]. A multiple dose escalation study of Hu5F9-G4, a humanized monoclonal antibody against the human CD47, for advanced solid malignancy or lymphoma (NCT02216409) indicated that it was well tolerated. Mild anemia was observed in some patients but can be managed without blood transfusion. Together, targeting innate immune checkpoint CD47 is a safe and excellent strategy as a monotherapy or in combination with other anti-cancer therapy [18]. Multiple clinical trials targeting macrophage have been initiated since 2014 for the treatment of several types of cancers [18].

Similar to CD47-SPIRα, Barkal *et al.* identified an essential role of major histocompatibility complex (MHC) class I in controlling the phagocytic function of macrophages through the expression of β_2_-microglobulin (β2M) by cancer cells. They found that leukocyte immunoglobulin-like receptor B1 (LILRB1) on the surface of TAMs binds to a portion of MHC-I on cancer cells, which inhibited the ability of macrophages to engulf the cancer cells. Blocking both MHC-I and LILRB1 pathways stimulated macrophage engulfment *in vitro* and *in vivo* and significantly slowed tumor growth in mice [19].

It was previously thought that PD-1 is expressed primarily on T cells and induces T cell exhaustion via the single immunoreceptor tyrosine-based inhibitory motif (ITIM) within its cytoplasmic tail [20]. When engaged with cancer cell PD-L1, the ITIM domain of PD-1 activates SHP2 to inhibit ZAP70 resulting in suppressing the activity of CD3/CD28 T cell receptor [21]. However, a recent study published in *Nature* by Gordon *et al.* reported the identification of PD-1-expressing TAMs [17]. In a mouse CT26 syngeneic mouse model, the authors found that 70% of TAMs express PD-1 on the cell surface compared with 2% and 1 % of that in the blood and spleen macrophages, respectively [17]. In the human colorectal cancer samples, the levels of PD-1^+^ TAMs are positively correlated with tumor malignancy. Functionally, the authors further demonstrated that PD-1^+^ TAMs are less capable of carrying out phagocytosis by (1) *ex vivo* phagocytosis assay using cocultures FACS-sorted PD-1^+^ and PD-1^−^ TAMs from CT26 tumors with GFP^*+*^*Staphy-lococcus aureus* bioparticles; (2) *in vivo* phagocytosis analysis using immunocompromised BALB/c Rag2^−/−^/γc^−/−^ mice engrafted with PD-L1-knockout CT26/YFP^+^ cells. The results from these two models suggested that tumor cells showing loss of PD-1/PD-L1 axis are phagocytized.

In addition, Gordon *et al*. further performed a bone marrow transplantation experiment to demonstrate the origin of the macrophage-induced tumor cell phagocytosis. To do this, donor bone marrow from RFP^+^ C57BL/6 mice was engrafted into irradiated host GFP^+^ C57BL/6 mice, which were then inoculated with MC38 colon cancer cells to the mice. After three weeks, they found that significantly higher fractions of PD-1^+^ TAMs were derived from donor RFP^+^ bone marrow, suggesting that PD-1^+^ TAMs originated from circulating leukocytes but not from resident immune cells in the host. They also showed that the combined therapy of HAC (anti-human PD-L1 small protein) and anti-CD47 enhanced anti-tumor efficacy and survival rate in a human DLD/GFP^+^ colon cancer xenograft mouse model with NSG mice. These well-executed studies provided important molecular insights into a potentially effective therapeutic strategy by elevating phagocytosis against cancers by targeting the PD-1 pathway with PD-L1 protein or anti-PD-1 and sensitizing CT26 cells to anti-CD47 therapy [17].

### An old dog with a new trick

**Fig. 1:**
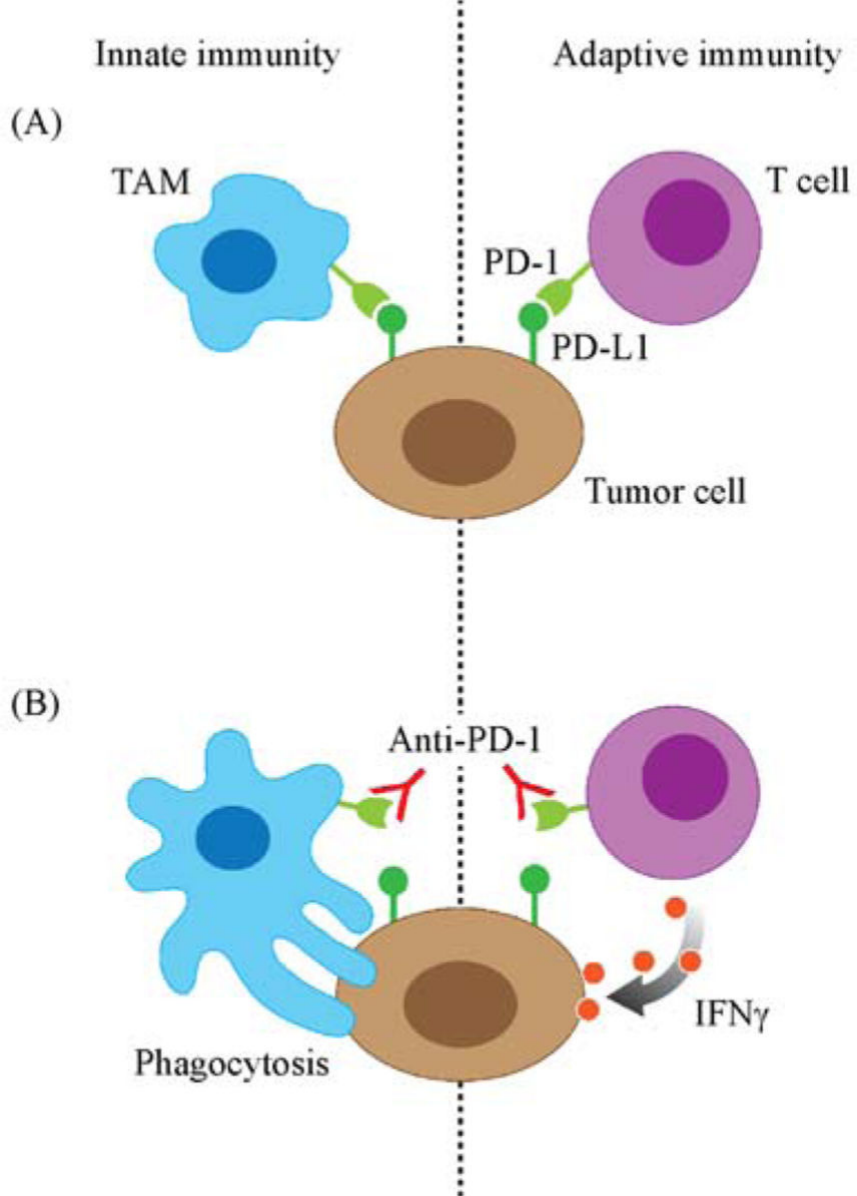
Anti-PD-1 therapy induces both TAMs and CD8^+^ T cell activity. (A) The expression of PD-1 on the tumor-associated macrophages (TAMs) and T cell inhibits antitumor immunity. (B) PD-1 antibody induces innate immunity by TAM phagocytosis and adaptive immunity by T cell cytolytic activity.

The study by Gordon *et al*. identifying a new mechanism of anti-PD-1, which can enhance the engulfment of cancer cells through TAM activation, has opened a new avenue toward the improvement of immunotherapy. Their findings suggested that the interaction between PD-L1 on tumor cells and PD-1 on PD-1^+^ TAM produces a “don’t-eat-me” signal that inhibits macrophage-mediated phagocytosis. Because macrophage-related immunity represents an innate immune response, this may partially explain why the efficacy of anti-PD-1 is more efficient than other types of immunotherapies as it can stimulate the phagocytosis of the cancer cells (Fig. 1). However, it remains unclear how much of the anti-PD-1 efficacy is attributed to phagocytosis. If the anti-PD-1-mediated antitumor effect requires phagocytosis, tumors with more TAM may respond better to anti-PD-1. In addition, proinflammatory cytokines or CD47 could serve as predictive markers for the anti-PD-1 therapy. It is likely that the innate immune response of TAMs induces phagocytosis of a particular type of cancer cells that share similar features to foreign pathogens. The engulfment of these cells by TAMs will (1) increase secretion of cytokines to attack T cell infiltration and subsequently (2) present tumor antigens for T cell activation.

Interestingly, the pro-inflammatory cytokine, IFNγ, which is secreted by CD8^+^ T cells, induces cytotoxicity against cancer cells. On the other hand, cancer cells are known to express high levels of PD-L1 on the cell surface to suppress the effector T cells via PD-1 [22]. This negative feedback regulation allows cancer cells to escape immune surveillance [23]. Likewise, a recent study suggested that another pro-inflammatory cytokine, TNFα, which is secreted by macrophages, induces stabilization of PD-L1 on cancer cells through COP9 signalosome subunit 5 (CSN5)-mediated de-ubiquitination [24]. If TNFα stabilizes cancer cell PD-L1 to engage with PD-1^+^ TAMs, the activated macrophages are subsequently inhibited. Therefore, the TNFα-PD-L1-PD-1 axis may be a new negative feedback loop that occurs between TAMs and cancer cells.

### Cooperation between innate and adaptive immunity

The findings by Gordon *et al.* also point to an important concept of the crosstalk between innate immune response and adaptive immunity (Fig. 1). Innate immunity represents a nonspecific defense mechanism that comes into play immediately when a foreign antigen appears in the body. Adaptive immunity refers to the antigen-specific immune response that requires more complex immune reaction for activation. Although the two immune systems crosstalk, it is not yet clear how they work with one another in the tumor microenvironment. To date, some studies have shown that CD8^+^ T cells play a critical role in mouse anti-CD47 blockade-induced tumor reduction even though the target is not directly on the T cells [25]. Depletion of CD8^+^ T cells diminishes the anti-tumor activity of mouse CD47 antibody in a syngeneic mouse model [25,26]. Moreover, IFNγ was significantly upregulated when mice treated with anti-CD47 [9], suggesting that while TAMs are engulfing cancer cells, the antigen presenting function of the macrophages induces CD8^+^ T cells to further eradicate cancer cells [18]. Similar to the notion, the presence of TAMs is critical for anti-PD-1 therapy. Because TAMs present tumor antigen for T cell activation, the more TAMs are present in the tumor area, the better the therapeutic outcome of anti-PD-1 [6]. On the basis of the findings by Gordon *et al.,* the presence of both innate and adaptive immune cells is critical for the anti-tumor activity. Molecules that are expressed on both TAMs and T cells may be useful to induce two types of the immune response against tumor progression.

### TAM, a two-edged sword

TAMs, mostly composed of M2 type macrophages, have been shown to provide a favorable microenvironment for tumor progression, angiogenesis, metastasis, and drug resistance in the hypoxic environment [27]. TAMs can suppress the CD8^+^ T cell immune response against cancer by directly interacting with T cells via the PD-1 pathway or by secreting immunosuppressive factors, e.g., IL-10 and TGF-β [28,29]. Clinicopathological studies often link the expression of TAMs with poorer disease outcomes [30,31]. The study by Gordon *et al.* showed PD-l^-^ TAMs can engulf cancer cells, adding new insight into the current anti-PD-1 therapy. However, some concerns still exist regarding the oncogenic function of TAMs. First, it remains to be determined whether activation of TAMs by anti-PD-1 for phagocytosis also promotes tumor aggressiveness or creates apoptotic insensitive tumor cells that escape T cells surveillance. In this regard, colony-stimulating factor 1 receptor (CSF-1R)-targeted therapy, such as RG7155 or PLX339, may be an alternative to reduce TAM polarization [32,33]. In addition, since TAMs produce the chemokine CCL22 to attract regulatory T cells and myeloid-derived suppressor cells to the tumor site, neutralization of CCL22 may reduce this potential adverse effect. If TAMs engulf cancer cells before its oncogenic activity, triggering TAMs self-apoptosis after phagocytosis can overcome the risk of TAMs activation. Thus far, more and more subsets of immunosuppressive cells have been identified, and are shown to be regulated in part by TAMs [34]. Harnessing TAMs-mediated tumorigenic phenotype may be more complicated than we expected.

### Other potential “don’t-eat-me” signals

Given the importance of TAMs and T cells’ immune reaction, we searched for other known T cell immune receptors that are also expressed on the macrophages and TAMs. Ideally, for molecules expressed on both T cells and TAMs, their therapeutic agents should be those that can stimulate both TAMs and T cell activation. According to the current published studies, we summarized 14 groups of immune checkpoints in Table 1. The PD-L1-PD-1 (Group 1), CD47-SIRPα (Group 6), and MHCI-LILRB1 (Group 8) are known to induce the “don’t-eat-me” signals that have been described above. Inhibition of these signaling can enhance both innate and adaptive immunity, so in-depth analysis should first focus on these three groups as their therapeutic agents may share similar efficacy and response profile. PVR-TIGIT (Group 4), B7–1/2 (Group 5), and OX40L-OX40 (Group 12), on the other hand, are expressed only on macrophages but not TAMs; thus, it is unclear whether they are involved in the function of phagocytosis. If so, how those activated macrophages migrate to the tumor area is an important question to be addressed. As for the other eight groups from the list, they are expressed on the TAMs, which is present in the tumor microenvironment to quickly act on tumor cells once activated. Importantly, some of their targeting agents are available and/or currently being evaluated in the clinical trials, e.g., anti-CTLA4, anti-VISTA, anti-TIM3, and anti-CD40, among others. It is therefore of interest to know whether those responders experience innate immune reactions during the treatment. Markers such as serum levels of CD163 (sCD163), the presence of CD68^+^ or CD163^+^ CD204^+^ macrophages in the tumor region can be used as indicator of macrophage or TAM activation following drug treatment [35].

### Future prospective

Uncontrolled outgrow by signaling deregulation is a hallmark of cancer. For many years, therapies targeting signaling pathway or protein activities have been promising strategies for many types of cancers. As a new type cancer therapy, immunotherapy, which enhances our immunity to fight against cancer, has been shown to be a relatively safe and tolerable choice. Thus, combination immunotherapy with chemotherapy or targeted therapy is an appealing strategy for combating the heterogeneity of tumors. To date, several therapeutic strategies have been successfully developed based on the understanding of the regulatory mechanism of immune checkpoints. Through the understanding of molecular regulation of immune receptors, TAM-mediated tumorigenesis may be limited.

In respond to IFNγ, cancer cells express PD-L1 to launch a feedback inhibition on T cells. Despite the understanding on the transcriptional regulation, post-translational modification of PD-L1 and its impact on cancer immunosuppression has emerged as an important mechanism for immune evasion. Based on the mechanisms underlying the post-translational regulation of PD-L1, many combinatorial strategies can be rationally designed with strong clinical value. Through EGFR and NF-κB signaling, we have previously reported several safe and effective combinations, such as gefitinib plus anti-PD-1 [36], curcumin plus anti-CTLA4 [24] and PARP-1 plus anti-PD-L1 [37] that may be worthy of being tested in clinical trials. Moreover, a study by Zhang *et al.* showed that stabilization of PD-L1 by CDK4/6 inhibitor sensitizes CT26 to anti-PD-1 [38]. CMTM6 [CKLF (chemokine-like factor)-like MARVEL transmembrane domain containing family member 6] maintains the membrane PD-L1 protein turn over, thereby reducing anti-tumor immunity [39,40]. The discovery of PD-L1 glycosylation also reveals that N-linked glycosylation protects proteins from GSK30-mediated degradation [36]. Monoclonal antibody targeting N192 and N200 glycosylation of PD-L1 can internalize PD-L1, which serves as a potential candidate for antibody-drug conjugate [41]. In this regard, identifying small molecule inhibitors that can effectively block posttranslational modification events may improve the effectiveness of current immune checkpoint blockades.

## Conclusions

The discovery of TAM PD-1^+^ by Gordon *et al.* shed new light on the inhibitory function of PD-1 beyond its role in T cell exhaustion. In addition to rejuvenating the cytotoxic T cell activity, anti-PD-1 therapy can also now stimulate TAMs to engulf cancer cells. The findings of that study suggested that coordination of innate and adaptive immune response is vital for anti-tumor immunity. Here, we identify several other immune receptors that are expressed on both TAMs and T cells. Therapeutic agents against these targets may induce a dual immune response and broaden the patient population for immunotherapy.

## Figures and Tables

**Table 1:** Immune checkpoints expression across three cell types categorized into different receptor-ligand pair groups

Group	Macrophages	T cells	Tumors	Suppressive/stimulatory	Localization	Targeted drug	References

1	PD-1 (CD279)		PD-L1	Suppressive	TAMs	Available	[17]
		PD-1	PD-L1	Suppressive			[42]
	PD-L1 (B7-H1; CD274), PD-L2 (CD273)						[43]
							
							
	PD-L1	PD-1		Suppressive	TAMs		[44]
	RGMb		PD-L2	Suppressive	TAMs		[45]
2	B7-H4				TAMs	Available	[46]
			B7-H4	Stimulatory			[47]
3	TIM3	GAL9		Suppressive	TAMs	Available	[48]
		TIM3	GAL9	Suppressive			[49]
4	PVR (CD155)	TIGIT		Suppressive		Available	[50]
		TIGIT	PVR	Suppressive			[51]
		CD226	PVR	Stimulatory			[52]
	PVR (CD155)	CD226		Stimulatory			[53]
		CD112R	CD112	Suppressive			[54]
5	B7–1 (CD80), B7–2 (CD86)					Available	[55]
							
		CD28	B7–1, B7–2	Stimulatory			[56]
		CTLA-4	B7–1, B7–2	Suppressive			
6	SIRPα		CD47	Suppressive	TAMs	Available	[57]
7	4–1BBL (CD137L)	4–1BB (CD137)	4–1BBL	Stimulatory	TAMs	Available	[58]
8	LILRB1		MHC I	Suppressive	TAMs	N/A	[19]
9	LRP1		CRT	Stimulatory	TAMs	N/A	[59]
10	RAGE		S100	Suppressive	TAMs	N/A	[60]
11	CD40		CD40L	Stimulatory	TAMs	N/A	[61]
		CD40	CD40L				[62]
12	OX40L	OX40		Stimulatory		Available	[63]
		OX40	OX40L				[64]
13	ICOSL	ICOS	ICOSL	Stimulatory	TAMs	Available	[65]
14	VISTA				TAMs	Available	[66,67]
		VISTA	VISTA-R				

N/A, not available.
